# Nutritional Deficiencies and Clinical Practices in Decompensated Cirrhotics With Hepatic Encephalopathy

**DOI:** 10.7759/cureus.25352

**Published:** 2022-05-26

**Authors:** Nishah N Panchani, Tyler Colvin, Mahmoud Aryan, Mohamed G Shoreibah

**Affiliations:** 1 Internal Medicine, University of Alabama at Birmingham, Birmingham, USA; 2 Gastroenterology and Hepatology, University of Alabama at Birmingham, Birmingham, USA

**Keywords:** thiamine deficiency, zinc deficiency, nutritional deficiency, hepatic encephalopathy, decompensated liver cirrhosis

## Abstract

Background

Hepatic encephalopathy (HE), a major complication of end-stage cirrhosis, is often associated with nutritional deficiencies. We aimed to assess the frequency in which vitamins and zinc were tested for and deficient in our cirrhotic population with HE.

Methods

We performed a retrospective chart review of 143 patients with decompensated cirrhosis that were seen in a hepatology clinic from January 2020 to May 2021. Patient demographics and decompensations were recorded. Vitamins and minerals that were evaluated included zinc, vitamin B12, folate, vitamin D, and thiamine. Continuous variables were reported as mean ± standard deviation and categorical variables were calculated as frequency percentages.

Results

Out of 143 patients, 73 were found to have HE. Out of 73, 33 were male, and the average MELD was 15.5 ± 6.3. 44% of patients had NASH cirrhosis, and 30% had alcoholic cirrhosis. Of the minority of patients that had their nutrient levels checked, 17/23 (74%) were deficient in zinc (<60 mcg/dL). 75% of patients were deficient in thiamine. 2/34 (6%) were deficient in folate (<5.9 ng/mL), 2/10 (20%) in vitamin D (<20 ng/mL), and 2/47 (4%) in B12 (<300 pg/mL).

Conclusion

Nutritional deficiencies are common in cirrhotics with HE. Further studies are needed to determine if routine testing and treatment for vitamin and Zinc deficiencies would have a positive impact on the clinical trajectory of HE.

## Introduction

Cirrhosis is a highly catabolic state with increased breakdown and underproduction of nutrients and proteins. The liver’s decreased ability to navigate through certain biochemical pathways, along with poor nutrition intake, protein malabsorption, gastrointestinal and urinary losses, and decreased synthesis all contribute to the poor nutritional status identified in decompensated cirrhosis [1]. Several studies have also shown increased morbidity and mortality in relation to malnutrition as cirrhosis progresses to end-stage liver disease [2].

 Hepatic encephalopathy (HE) is a very prevalent presentation of decompensated cirrhosis and is associated with high morbidity and mortality. The liver’s decreased ability to effectively convert ammonia to urea leads to build-up in the brain contributing to the symptoms of HE [3]. Nutrients like zinc play a crucial role in the metabolism of ammonia and are often low in patients with liver disease [4]. Thiamine deficiency also contributes to encephalopathy by means of oxidative stress, increased inflammatory cytokines, impaired mitochondrial pathways, and overall brain lactate accumulation [5]. Similarly, approximately half of the body’s cobalamin is stored in the liver, and B12 deficiency can present as neurological symptoms [6].

Several studies have looked at micronutrient deficiencies in liver cirrhosis and methods of effectively identifying those patients that may be at higher risk of these losses [7,8]. Some studies have also shown that educating patients and obtaining appropriate nutritional counseling helped in reducing admissions [9]. However, when it comes to clinic visits in the outpatient setting, there is a lack of consistent guidelines that highlight when to screen for malnutrition.

Given that malnutrition is a common problem in cirrhosis, we aimed to identify the frequencies at which zinc, thiamine, vitamin B12, and other nutrients were evaluated at our institution, particularly in patients with a diagnosis of HE secondary to decompensated cirrhosis. We also aimed to evaluate whether patients with HE were more likely to be hospitalized compared to patients without HE, as well as the nutritional status of these patients.

## Materials and methods

Study design and patient population

We performed a retrospective chart review of patients with decompensated cirrhosis seen in an outpatient hepatology clinic from January 2020 to May 2021 at a tertiary, academic medical center. The study was approved by the institutional review board. All data was collected from a secure electronic medical record and stored in a protected excel sheet. 

Patients with compensated cirrhosis were excluded from the study. Patients above the age of 18 and with decompensated cirrhosis met the inclusion criteria for the study. Decompensated cirrhosis was defined as the presence of cirrhosis with either HE, volume overload, ascites, esophageal varices, variceal bleed, hepatocellular carcinoma, spontaneous bacterial peritonitis, jaundice, pruritus, or gastric antral vascular ectasia. A total of 143 patients were included in the study.

Data collection

Patient baseline demographics consist of age, sex, and origin of cirrhosis in addition to manifestations of decompensated cirrhosis including volume overload, ascites, esophageal varices, variceal hemorrhage, HE, and transjugular intrahepatic portosystemic shunt (TIPS) placement was recorded. Patients with HE that were well controlled on medications were not recorded as having active HE. Laboratory data associated with the model for end-stage liver disease-sodium (MELD-Na) score were also measured as were lactulose and rifaximin use. Hospitalizations during this time period were recorded along with the presenting diagnosis. Vitamins and minerals levels including B12, thiamine, zinc, folate, and vitamin D were recorded from January 2018 onward and included both inpatient and outpatient testing of whole blood.

Statistical analysis

Baseline descriptive characteristics of our cohort were represented by a mean ± standard deviation (SD) for continuous variables as well as a frequency percentage for categorical variables. With respect to HE as well as other manifestations of decompensated cirrhosis, a stratified frequency percentage of those who were tested for various vitamin and mineral levels as well as of those who were deemed deficient in such vitamins and minerals was reported. Chi-squared analysis to compare outcome variables in those with HE versus those without HE was performed.

## Results

Our entire study population consisted of 143 patients with an average age of 57.81 ± 11.40 and 71 (50%) were male. Etiology of cirrhosis included 49 (34%) patients with nonalcoholic steatohepatitis (NASH) cirrhosis, 43 (30%) patients with alcoholic cirrhosis, 14 (10%) patients with hepatitis C virus (HCV) cirrhosis, and the remaining 37 patients (26%) with other causes or a mixed etiology of cirrhosis. The mean MELD-Na score of the entire cohort was 13.80 ± 5.79. Fifty patients had Child-Pugh score A, 64 had B and 23 patients had Child-Pugh C. As far as cirrhotic decompensations were concerned, 73 (51%) had active HE, 58 (41%) had volume overload, 52 (36%) had ascites, 34 (24%) had esophageal varices, 20 (14%) had a history of variceal hemorrhage, 15 (11%) had hepatocellular carcinoma, and 14 (10%) had TIPS. 

Stratified analysis of those with HE depicted 33 (45%) males with a MELD-Na score of 15.5 ± 6.3, mean albumin level of 3.35 ± 0.6 g/dL, and overall etiology of cirrhosis including NASH cirrhosis (44%), alcoholic cirrhosis (30%), hepatitis B or C alone or in combination with alcohol (13%) and other (15%). For the management of HE, 27 (48%) were on lactulose, eight (11%) were on rifaximin, and 35 (48%) patients were on both lactulose and rifaximin. TIPS placement was found in eight (11%) of these patients (Table 1).

**Table 1:** Patient demographics and variables of the entire cohort compared to those with hepatic encephalopathy displayed as frequency percentages

Variables	Patient Data N, (%)	Hepatic Encephalopathy Patients N, (%)
Total	143, (100%)	73, (51%)
Age		
<65	99, (69.3%)	48, (66%)
Sex		
Male	71, (49.6%)	33, (45%)
Female	72, (50.4%)	40, (55%)
Cirrhosis Cause		
NASH	49, (34.3%)	32, (44%)
Alcohol	43, (30%)	22, (30%)
HCV	14, (9.8%)	4, (5.5%)
PSC/PBC	4, (2.8%)	1, (1.4%)
HBV	3, (2.1%)	2, (2.7%)
Other	30, (21%)	12, (16%)
Decompensations		
Volume	58, (41%)	32, (44%)
Ascites	52, (36.3%)	24, (33%)
Varices	34, (23.8%)	17, (23%)
HCC	15, (10.5%)	5, (6.8%)
Other	7,(4.9%)	2, (2.7%)
MELD Score		
MELD<20	119,(83.2%)	55, (75%)
TIPS	14, (9.8%)	8, (11%)
Hospitalizations	64, (44.8%)	44, (60%)
HE primary	20, (14%)	20, (27%)

**Figure 1:**
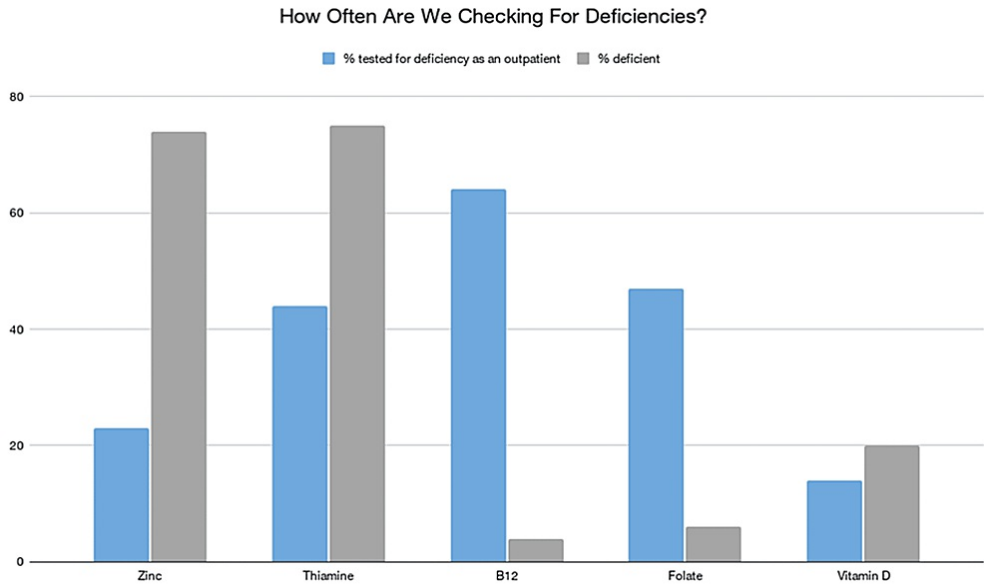
Frequency percentages of vitamins and zinc levels that were checked in an outpatient hepatology clinic compared to the percentages of patients that were deficient.

During their clinic course, only 23 patients were tested for zinc deficiency, of which 17 (74%) were deemed deficient (<60 mcg/dL). Low zinc levels were significantly correlated with higher Child-Pugh scores; nearly 54% of patients with zinc deficiency had a score of B, compared to 25% patients with C and 20.8% with a score of A (p=0.039). Exactly 32 (44%) HE patients had their thiamine level tested of which deficiency was found in 24 (75%) patients, with 11 (34%) being at a low (8-30 nMol/L) level, and 13 (41%) being at a severely low level (<8 nMol/L). B12 level was tested in 47 (64%) patients where deficiency (<300 pg/mL) was found in two (4%) of these patients. Furthermore, 34 patients had their folate level tests of which two (6%) were deemed deficient (<5.9 ng/mL). Only 10 patients were tested for vitamin D deficiency where two (20%) were found to be deficient (<20 ng/mL) (Figure 1).

Regarding outcome variables, the chi-squared analysis showed that patients with HE are statistically significantly more likely to be hospitalized within a year of their clinic visit, as compared to those without HE (62% vs 27%, p-value<0.001). We also found that patients with an admitting diagnosis of HE did not have their zinc levels checked 50% of the time, whereas 45% of patients did not have their thiamine levels checked.

## Discussion

Identifying nutrient deficiencies in decompensated cirrhosis is particularly crucial to disease management. HE can be a very debilitating side effect of cirrhosis, however overlapping malnutrition can propagate symptoms and worsen presentation. Our study showed that a large percent of HE patients were deficient in zinc and thiamine, two important micronutrients that aid in processing and metabolizing toxins. We were also able to demonstrate that patients with HE were more likely to be hospitalized compared to their non-HE counterparts. Furthermore, patients that were hospitalized with HE as one of the admitting diagnoses were not screened 50% of the time for vitamin or zinc deficiencies to rule out micronutrient inadequacy. Our data revealed that micronutrients are not being tested for on a regular basis, even if the patient presents with signs or symptoms of worsening HE despite being on a bowel regimen. Our population mostly had normal vitamin B12 levels which are likely due to the known phenomenon of increased circulation and leakage of cobalamin secondary to decreased uptake by the liver [6].

Several studies have also observed impressive deficiencies in zinc. Llibre-Nieto et al. noted about 85% prevalence of zinc deficiency; however, none were identified with B12 deficiency similar to our study. Studies have particularly shown that patients in Child-Pugh B or C are at a higher risk of developing zinc deficiency as well as other nutrient and caloric deficits [7,8,10]. Overall, there are no set guidelines to date that suggest checking for or monitoring patients with cirrhosis for micronutrient deficiencies in the outpatient setting. The results of our study point to the concept of suboptimal nutrition in our decompensated cirrhotic population and maybe a catalyst to change in practice guidelines in the future.

Our study is limited by the small sample size and does not look into complications and outcomes other than hospitalizations related to HE. Micronutrient levels were infrequently obtained by providers, and repeat micronutrient levels after appropriate supplementation were not available for most patients in this retrospective study.

## Conclusions

In conclusion, our study showed that micronutrient levels were not frequently checked in our outpatient cirrhotic population with HE, and those that had their levels checked were largely deficient. Further studies need to be conducted to identify whether routine checking of micronutrient deficiencies, particularly zinc, thiamine, and B12, have any benefit when it comes to morbidity and mortality in decompensated cirrhosis and HE.
